# The Psychological Assessment of Clerics

**DOI:** 10.1007/s11089-017-0784-y

**Published:** 2017-07-14

**Authors:** Alexander Jack, Daniel T. Wilcox

**Affiliations:** 1 Centre for Family and Forensic Psychology, Division of Psychiatry and Applied Psychology, School of Medicine, University of Nottingham, Nottingham, NG81BB UK; 2 Wilcox Psychological Associates, 55 Raddlebarn Road, Selly Oak, Birmingham, B296HQ UK

**Keywords:** Psychological assessment, Religious organizations, Secular, Suitability, Risk

## Abstract

The psychological assessment of novice and experienced clerics is an important component of ordination, suitability, and risk evaluation to ensure that representatives of religious organizations are equipped, motivated, and safe for a life commitment to a faith vocation. It is the authors’ opinion that such assessments should be conducted by skilled psychologists with expertise that covers occupational, clinical, and forensic domains. Further, the authors emphasize the importance of an objective and secular assessment to better inform the church about its role and responsibility for the oversight and spiritual development of the cleric. A thorough psychological assessment should incorporate a multimodal approach to information gathering, which includes a comprehensive review of background information and medical records, a personal history interview, a mental status examination, and administration of relevant psychometric measures and assessment tools. We also advise that, upon completion of the assessment, the requesting religious organization should be offered the opportunity to meet with the evaluating psychologist to discuss suitability issues and, if necessary, risk management planning.

## Introduction

The sacrifices and commitments involved in dedicating oneself to a life of piety and religious servitude are considerable. An individual preparing for ordination must be psychologically ready to uphold the religious vows of his or her chosen faith vocation. As a result, any religious organization is well advised to fully assess the suitability of prospective clerics, given the individual, organizational, legal, and reputational consequences of making ill-suited choices. In addition, ongoing commitment can, at times, wane amongst established clerics for identifiable clinical or other reasons. Further, forensic risk issues can arise as matters of concern in the lives of clerics, and all of these issues will require careful evaluation.

There is a rich diversity in religious belief and expression in the United Kingdom. However, the present paper predominantly relates to Christian organizations. There are approximately 340 Christian denominations in the United Kingdom, with over thirty-five thousand ministers and priests (Brierley 2011). The 2011 census reported that almost 60% of the U.K. population considered themselves to be Christian (Office for National Statistics 2011). As such, Christian religious leaders continue to play an important societal role, representing over thirty million self-professed Christians and offering spiritual guidance to many others who are less demonstrative or clear about their religious convictions.

In relation to addressing identified needs across this population, McGlone et al. (2010) note that the Catholic Church in the United States frequently seeks assistance from mental health professionals to conduct assessments—often through the services of psychologists. This paper will discuss the utility of psychological evaluation in providing an underpinning structure for these various types of assessments to complement the ongoing evaluation of spiritual concerns, for which the church rightly holds full accountability. Integral to this approach, we espouse the necessity of an evidence-based framework for evaluating occupational, clinical, and forensic concerns to assist in establishing whether an individual is, or remains, suited to a life of service to the church. We advise that the requirement of secular objectivity should be a key aim of these aspects of assessment as, collectively, decisions about these matters impact individuals, families, communities, and the wider society. Accordingly, we consider that the standards for assessment must reflect the same levels of rigor and expertise that are expected within clinical and legal settings.

## Reasons for referral

In our experience, prospective and practicing clerics may be referred for psychological assessment for a number of reasons. The evaluating psychologist might be instructed to assess factors relating to the referred cleric’s personal effectiveness and psychological well-being, for example, giving a view as to whether a Catholic novice is suited to fulfilling the requirements of his role. However, in other circumstances, it may be necessary to assess the extent to which a person’s continuing life as a cleric is achievable and indeed desired by the individual being evaluated. Importantly, a third reason for referral may be to assess any risk that the cleric may pose to others.

Nevertheless, and irrespective of the specific focus of the psychological evaluation undertaken, some key areas, including affective maturity, interpersonal skills, empathic abilities, and psychosexual development, are likely to be assessed (McGlone et al. 2010). Additional issues typically explored are sexual experience, orientation, and inclination; capacity for maintaining celibacy (for Catholic priests and nuns); history of substance misuse; attitude to authority; level of insight; ability to communicate effectively; and capacity for leadership in representing the values and doctrines of the Church (McGlone et al. 2010).

### Referral for spiritual assessment

We note that many psychological screenings or risk-related evaluations of clergy or novices have a spiritual assessment component. However, in our judgement, examination of this issue is not within the purview of a psychological evaluation. Nevertheless, some related features, including motivation, work satisfaction, and a number of person-specific traits, may have an impact upon this area of inquiry. We note that some measures, such as the Attachment to God Inventory (Beck and McDonald 2004) and the Spiritual Well-Being Scale (Paloutzian and Ellison 1999), may provide information that will be of assistance to the cleric-subject’s spiritual supervisor, and we would, therefore, consider administering such measures. However, it is our view that those who oversee the cleric’s work and spiritual development are best placed to make judgments in this area. As such, the evaluating psychologist may play an advisory and liaison role, offering empirical findings in support of the Church’s spiritual assessment of the novice or cleric.

### Vocational suitability assessment

Although this area may be thought to be more appropriate for an occupational psychologist to assess, we consider that important clinical and forensic factors may present that make it advisable to augment these latter skills with the acquisition of a strong and informed base of knowledge about the demands and expectations placed on the working lives of clergypersons. As an example in relation to the assessment of clerics, some notable areas of vulnerability are observed that appear to be, to some degree, vocation specific. For example, research by Knox et al. (2005) indicated that Catholic priests reported seven times more depression than the general population of adults, as well as low levels of satisfaction with their work. Relatedly, many priests report feeling overwhelmed by their vocation (Rossetti and Rhoades 2013), with key contributing factors including a sense of isolation and lack of social support (Virginia 1998).

Lewis et al. (2007) reported that stress associated with spiritual and religious leadership responsibilities can bring about increased rates of ‘burnout’ among clerics. They also reference the popular myth that such job exhaustion is unlikely to affect priests owing to the chosen nature of their vocation. Nevertheless, stress levels beneath the surface are often found to be both prolonged and intense among religious and spiritual leaders. Hypothesizing about this, Lewis et al. (2007) considered that the increasing challenges posed by adhering to traditional doctrines in an increasingly modern and secular world may be a significant contributing factor.

In terms of determining the ‘right fit’ for entering clergy and demonstrating their ability to successfully maintain positive work attitudes, Francis et al. (2009) noted that clerics who were introverted and more thoughtful were likely to report less resilience over time and more work-related psychological challenges when compared with their extroverted and more emotionally oriented colleagues. Although this observation does not reflect any judgement about these character types in general, it does appear to highlight the need to consider broader aspects of psychological makeup when making decisions about the selection of individuals who may more likely succeed in a religious vocation. The use of psychometric measures can take account of these various factors when examining personality features and can offer a helpful further dimension to assessment.

### Referral for risk assessment

When instructed to conduct a psychological assessment, risk issues may present as a prominent concern, with allegations of sexual harm more often coming to the fore in clerical assessments than physical or emotional abuse issues on their own. Notably, the impact of sexual abuse within the Catholic Church has been considerable, both in terms of the great harm caused to victims and the deeply damaging effect this has had on the reputation and standing of the church. Terry (2008) has noted that almost $2 billion was spent by the Catholic Church in the United States between 1950 and 2002 on treatment, legal fees, and compensation to victims.

It is beyond the task of this paper to fully explore factors that may contribute to sexual abuse risk within the church and Christian organizations more generally, though the reader may find Pilgrim (2012) to be of assistance in terms of detailing these matters. Relatedly, there is increasing pressure on religious institutions to confront abuse directly and proactively. Therefore, experienced psychologists with relevant forensic training and expertise in the assessment of sexual offending have begun to take a more prominent role in such evaluations.

Broadly, sexually harmful behavior has been the subject of considerable research over the past 30 years (see Beech et al. 2009), though clerics who sexually abuse have, until recently, been less closely examined than many other populations (Plante 1999). Nevertheless, a literature base has gradually taken shape, and Perillo et al. (2008) have noted similar risk factors that are relevant to both clerical and non-clerical abusers. Haywood et al. (1996) reported that clerics convicted of sexual offending tend to be older, better educated, and single when compared to other sex offenders. Further, Haywood and colleagues noted that clerics who offended were more likely to target fewer victims and to direct their attention to older adolescents in comparison to non-clergy sexually abusive controls, who proportionally abused more victims and were more likely to target younger children.

We assert that the sexual abuse of parishioners (non-minors) by clerics may best be viewed as a professional sexual misconduct offense. In relation to this, we note that this offense entails the exploitation of the inherent power and status differential between the perpetrator and the victim (Funmilayo et al. 2015). We note that the position of a priest or minister is one of significant authority, particularly amongst his or her parishioners. Further, a sexual advance by such a cleric would likely produce significant dissonance for a parishioner, who might be fearful of disobeying the cleric or occasioning his or her disapproval. As such, this might heighten their vulnerability to manipulation and victimization. Indeed, this would be a particularly challenging and distressing experience for a parishioner who might, in normal circumstances, seek guidance and support from the Church to extricate him or herself from any such potentially threatening situation.

Pilgrim (2012, p. 408) writes that the “Catholic culture of spiritual authority, invested in the visible clothing (‘cloth’) of religious staff, meant that children in these settings certainly experienced *a particular form of spiritual entrapment. . . .* Victimized Catholic children [believed] that religious personnel were God’s representatives on earth. The religious cloth and learned rituals of piety and obedience meant that conflation was experienced for some of the victims between an adult-dominated moral order and God’s expectations of obedience and faith.”

In our experience, within the psychological assessment process, a range of factors may emerge that are associated with the cleric’s sexually harmful behavior. In some instances, there may be notable indications that the cleric has made efforts to avoid offending, though their self-management abilities have proven ineffective; in other cases, the offending may be instrumental or even predatory in nature (Ward and Siegert 2002). This makes it all the more important to systematically examine the apparent motivations in the offense account. Further review might prompt a line of questioning around, for example, why the individual chose to pursue a religious vocation. Relatedly, research has indicated that sex offenders are overrepresented in professions that direct their efforts towards working with children and young people (Colton et al. 2010; Moulden et al. 2007). As such, motivation to join the priesthood might be influenced by the extent to which the individual is ego-dystonic (distressed) or ego-syntonic (comfortable) about having deviant sexual urges. As Pilgrim (2012) notes, celibacy is not a risk factor per se. However, the priesthood can be attractive to an ego-dystonic individual wanting to escape sexual temptation or to an ego-syntonic offender wishing to more freely access victims.

The above issue is an important consideration, and offenders can vary in the degree to which they may be conflicted or emotionally at ease or resolved about their deviant interests. Nevertheless, this matter has great bearing on the assessment of future potential risk and treatability, as well as insight into the referred individual’s commitment to his or her role as a cleric. Relatedly, although detailed questioning about sexual functioning and behavior can be awkward for both interviewer and interviewee, such discussion must, in our view, form an integral part of any sexual risk assessment (Leonard and Donathy 2017), irrespective of whether faith-related sanctions regarding, for example, celibacy, masturbation, or other sexual matters are explicitly reflected in mandated church doctrine.

### Referral for assessment of homosexual tendencies

It might be the case that the evaluating psychologist is asked to assess the referred individual’s proclivity towards homosexuality. Indeed, Songy (2007) identified this to be an important aspect of the assessment of clerics. However, as with heterosexuality, if, as in the Catholic Church, a vow of celibacy is taken, then sexual orientation would seem to be a matter of little consequence so long as the cleric’s pursuit of the priesthood was not made to pursue opportunities for gay sexual liaisons. However, it is our view that the pathologizing of normal (homosexual) sexual behavior, on the grounds of religious doctrine, is ethically questionable and, as such, is outside of the domain of the psychologist. However, psychological adjustment relating to inhibiting active homosexuality may be a consideration (Kappler et al. 2013). Again, we emphasize the importance of secular and objective assessment to impartially inform the church’s decision-making.

## The process of assessment

A comprehensive psychological assessment is multi-faceted to enable the evaluating psychologist to gain a robust and thorough understanding of the referred individual (Wilcox 2000). To assist in this effort, we recommend an approach that incorporates a review of pertinent background documentation, a series of interviews, the implementation of carefully considered psychometric assessment tools, and, if appropriate, when greater levels of dissimulation seem apparent—as with sexual risk assessment (Wilcox 2009, 2013)—the polygraph may be helpful to augment and enhance the evaluation process.

Conducting a psychological assessment and, in particular, an evaluation examining perceived concerns about risk issues will almost invariably cause the referred individual to experience considerable apprehension, especially at the outset of the assessment. Such anxiety can impact the individual’s ability to engage in an open and honest discussion of events, thoughts, feelings, and behaviors relevant to the evaluation. Further, it is not uncommon for individuals in these circumstances to attempt to positively misrepresent themselves. This occurs for a variety of reasons—for example, as a strategy to protect self-esteem or to avoid the perceived negative consequences of being more fully disclosing (Wilcox and Gray 2017). To address these hurdles, we consider it important at the outset to broadly outline with the client the assessment approach that will be taken and to discuss the specific aims related to the referral prior to commencing the evaluation. In our experience, this can help to establish rapport and affirm that the individual is being assessed, not judged. Without doubt, a thorough review of all relevant available documentation will facilitate this process. The evaluating psychologist should seek to form a collaborative relationship (Ware and Harkins 2015), whereby the two parties work together to clarify issues and thoroughly assess the key areas being examined. We note that Thornton et al. (2000) found that denial and minimization of problem behavior could be reduced through an open, encouraging, and supportive approach to engagement.

The existing literature on clerical assessment confirms the relevance and benefits of adopting this orientation within the assessment process. McGlone et al. (2010) stressed the importance of an evaluation that considers strengths as well as pathological features in the referred individual and incorporates a battery of appropriate psychometric measures. Further, when sexually harmful behavior is an identified area of concern, we endorse the undertaking of a detailed psychosexual history. Plante and Boccaccini (1998) outline an example of such an assessment protocol to be used with clerics.

### Case documentation and medical record review

A psychological assessment normally begins with the receipt of a letter of instruction compiled by the referring party that gives clear direction for the assessment process and sets out the specific areas to be assessed. The instructions should be accompanied by all pertinent reports and documents, including, when possible, medical records for the referred individual. Review of such materials enables the performance of a more comprehensive and integrated assessment. This provides an opportunity to cross-reference information gathered and give greater shape and direction to the interview process. It is at the discretion of the evaluating psychologist to decide whether to undertake a thorough document review before the first client appointment. However, it is our opinion that a full examination of case documents prior to the first meeting can interfere with the data gathering stage of assessment, as it may, paradoxically, diminish the examiner’s understanding of the client’s normal behavior within the context of the forensic assessment. Therefore, a thorough review of the case documentation may result in a potentially missed opportunity, as later cross-referencing of information often produces valuable insights into the client’s behavior concerning what was omitted, included, or misrepresented in the initial interview. This can then assist greatly in shaping further interviews to pull together the various strands of the assessment, addressing inconsistencies and facilitating the production of an integrated and more comprehensively informed final report.

We consider that a thorough review of all relevant background information contributes to the best assessment results, but this is not always achievable. Typically, in legal or statutory forensic investigations, reports are available (from courts, local authorities, solicitors, social workers etc.). However, this is less likely to be the case with clerics, who may have had no prior contact with forensic systems and services. In such cases, the gathering of accurate information during assessment is more challenging, though still very important.

### Personal history interview

The personal history interview is a semi-structured interview designed to address the individual’s background, including family history, important life events, and past involvement with authorities. Further, an understanding of the individual’s experiences of education, work, and relationships is systematically explored during the interview and contextualized in relation to the issues considered pertinent to the referral (Wilcox and Gray 2017). This approach is considered appropriate when assessing clerics (McGlone et al. 2010; Plante and Boccaccini 1998).

We recommend conducting the personal history interview at the outset of the assessment because it provides an opportunity to develop rapport and a broad understanding of the client without specific reference to the perceived presenting problems. Nevertheless, if an individual has experienced trauma or significant distress, this exercise may still be a challenging experience, which necessitates a sensitive approach and a willingness on the part of the assessor to demonstrate appropriate patience, concern, and empathy. As such, in dealing with these kinds of issues, it is important for the evaluating psychologist to be mindful of *process* issues as well as *content*. This means considering how the interviewee recalls and presents information, and such clinical attunement can enhance the assessor’s understanding of what happened as well as of how the individual has given personal meaning to these events. This can also demonstrate to the client a level of concern for him or her as a person that will facilitate openness and fuller engagement in the assessment.

### Mental status screening

The mental status examination is an important adjunct to the assessment as it allows the evaluating psychologist to gain a greater understanding of the individual’s current functioning and present ‘state of mind.’ Within the context of this interview, various features are examined, including the client’s appearance and interpersonal style, hygiene and self-care, as well as employment of maladaptive coping strategies including alcohol misuse, illegal substance use, heightened emotional reactivity, social withdrawal, etc. Difficulties across these domains might reflect adjustment challenges and personality features of relevance to assessment and treatment. Such issues may have a bearing on global functioning and an impact upon lifestyle and attitudes to work, as well as potential risk when that is being assessed.

Such information can often provide insights and, in the case of sexual harm, identify concerns, informing the development of intervention strategies targeting the ‘risk,’ ‘responsivity,’ and ‘need’ principles of treatment (Bonta and Andrews 2007). Employing these interviewing and assessment tools, the evaluating psychologist can systematically collect information regarding social and emotional challenges and sexual attitudes including preoccupation and distorted pro-offending thought patterns, as well as deviant interests and self-control deficits.

With regard to clergy specifically, McGlone et al. (2010) highlighted a focus during the interview process that carefully explores several factors particularly pertinent to psychological assessments conducted with priests in the United States (see Table 1); we believe that the mental status evaluation and personal history interview provide a helpful framework for examining these issues and their relevance to various types of clerical assessment.

**Table 1:** *Considerations When Assessing Clergy (*McGlone et al. 2010 *)*

Affective Maturity
Interpersonal Skills
Capacity for Empathy
Psychosexual Development
Sexual Experience
History of Substance Abuse
Sexual Orientation or Inclination
Capacity to Live Celibate Chastity
Manner of Dealing with Authority
Level of Self-Knowledge
Capacity for Self-Reflection
Ability to Communicate Effectively
Capacity for Growth and Conversion
Ability to Grasp Abstract Questions
Capacity for Leadership
Decision-Making Skills
Ability to Grasp Practical Questions
Capacity for Critical Thinking

### Application of psychometric measures

The employment of psychometric testing, in addition to document review and clinical interviews, offers the best opportunity to produce a comprehensive psychological report (Wilcox 2000). Over time, a skilled psychologist will become experienced in employing a variety of standardized psychometric measures that can complement information gathered from other sources or even provide additional insights concerning relevant topics. Almost invariably, psychometrics afford an opportunity to broaden and enhance the assessment base, bringing specific areas of enquiry into finer focus and enabling important comparisons to be made between the thoughts, feelings, behaviors, and risk-relevant factors of the individual being assessed in relation to his or her peers. Importantly, this exercise has particular merit when evaluating actuarial, dynamic, and acute risk levels. The use of psychometric tools often enables the acquisition of additional information in a manner that is easier and less threatening than various aspects of the interview process for the individual being assessed.

## Use of the polygraph

For evaluating sexual risk, we find the polygraph instrument to be of particular benefit, especially when reported concerns are considered substantial through legal involvement and ‘hard’ evidence is, at that stage, limited. The polygraph is designed to monitor and record changes in an individual’s physiological reactions in response to various stimuli presented by the polygraph examiner. Specifically, the instrument gathers data concerning changes in respiration, blood pressure, and galvanic skin response (e.g., perspiration). Although often characterized as a ‘lie detector,’ the polygraph’s utility, in our experience, is most apparent in terms of truth facilitation because considerably more information is gathered during a polygraph examination than a simple judgment as to whether someone is being truthful or deceitful.

The client is systematically questioned whilst simultaneously comparing physiological responses to the issues being raised. Significant variations in responses are considered to reflect indications of whether the individual is being truthful or deceptive in replying ‘yes’ or ‘no’ to the pre-agreed items. Some items are directly relevant to the assessment, some serve as comparison items, and some, such as items confirming the client’s name, age, etc., are irrelevant. The polygraph is considered to be much more accurate in terms of distinguishing between truthful and deceptive responses than even the most astute professionals who do not employ this technology. The employment of polygraphy with sex offenders, both for initial evaluation, with the agreement of the individual at the arrest stage, and, compulsorily, at the post-conviction stage, has become accepted practice in the United Kingdom owing to the perceived significant assistance it provides in assessing, treating, and monitoring sexual offenders (National Offender Management Service 2015; Offender Management Act 2007; Wilcox and Donathy 2008; Wilcox and Gray 2012). Clearly, the use of the polygraph, or indeed any other assessment tool, must be carefully weighed in relation to the perceived and agreed-upon requirements of the requested psychological report.

Detailed examination of the employment of the polygraph to evaluate sexual risk is beyond the scope of this paper; see Wilcox (2009), 2013) for a thorough review. Although still a subject of controversy, the utility of the polygraph is considered to be substantial amongst professionals working in the field, and its use as an adjunct to sexual psychological risk assessment has become established practice in this context in the United Kingdom (Gannon et al. 2012, Gannon et al. 2014; Grubin 2006, 2010; Wilcox and Donathy 2014).

## Summary and conclusions

In our view, the use of psychological assessment methods can have a significant positive impact on the evaluation of clergy. This may occur in determining their suitability to take up such a vocation or to remain committed to a religious life. Beyond assessing an individual’s capacity for devoting their life to the Church, focal assessments are also within the purview of psychological reporting, as with the evaluation of risk issues wherein we endorse a decidedly secular approach to ensure objectivity in reporting. We support the use of a wide range of the assessment tools referenced in this article and consider that upon concluding the assessments, meetings with the senior cleric and the child protection officer (“safeguarding officer” in the United Kingdom) for the organization is of key importance for providing input into an agreed-upon plan of action.
